# Dietary supplement use in high-intensity functional training (HIFT) athletes by competition level

**DOI:** 10.1080/15502783.2025.2550168

**Published:** 2025-08-26

**Authors:** Jack Livingston, Sofea Smith, Kworweinski Lafontant, Claudia Gonzalez, Michelle Da Silva Barbera, Linh Nguyen, Susan Kampiyil, Jeffrey R. Stout, David H. Fukuda

**Affiliations:** University of Central Florida, Physiology of Work and Exercise Response (POWER) Lab, Institute of Exercise Physiology and Rehabilitation Science, Orlando, FL, USA

**Keywords:** Dietary supplement, HIFT, recovery, rehydration

## Abstract

**Background:**

Dietary supplements (DS) are used by athletes to aid sport-specific performance and recovery. High Intensity Functional Training (HIFT) is a unique sport with muscular strength/power/endurance, aerobic endurance, and rehydration concerns. While a multitude of DS may be beneficial for HIFT athletes, yet usage patterns unclear.

**Methods:**

Forty-nine HIFT athletes (24 women, 25 men; Age = 37.8 ± 10.5 years; BMI = 25.7 ± 3.7 kg/m^2^) completed a supervised Qualtrics cross-sectional survey. Regarding competition level (CL), Athletes were classified as noncompetitive, local, regional, or national based on previous HIFT competition experience. Participants reported DS use and provided open-ended rationales for use or discontinuance. Data are presented descriptively; no inferential statistics were conducted due to low sample sizes between CLs. Two coders performed inductive thematic analysis via consensus to reveal initial codes and final themes.

**Results:**

The distribution of CL were: noncompetitive = 12 (25%), local = 30 (61%), regional = 4 (8%), national = 3 (6%). Most participants reported using DS (*n* = 46, 94%) with caffeine (*n* = 35, 76%) and electrolyte drinks (*n* = 35, 76%) as the most favored supplements. Whey/casein protein (*n* = 33, 72%), creatine (*n* = 32, 70%), and multi-vitamins (*n* = 22, 48%) rounded out the top five DS. These were similar among all CL, except for magnesium and multi-vitamins being used among all three national-level athletes. Prevalent reasons for using DS were general health (*n* = 23, 50%), recovery optimization (*n* = 19, 41%), and rehydration (*n* = 13, 28%). Half of the athletes (*n* = 23) had discontinued at least one supplement, chiefly because of lifestyle changes (*n* = 7, 30%), perceived lack of benefit (*n* = 7, 30%), or cost (*n* = 3, 13%); adverse effects were rarely the reason for discontinuation (*n* = 1, 4%).

**Conclusions:**

Across competition tiers, HIFT athletes most commonly rely on caffeine, electrolyte drinks, creatine, dairy-based protein, and multivitamins. While we were limited by a small sample size, the varying motives for DS use suggest that practitioners should assess the rationale behind DS use for HIFT athletes before providing evidence-based advice.

